# Misdiagnosis of persistent left superior vena cava with unroofed coronary sinus as a coronary sinus‐type atrial septal defect

**DOI:** 10.1002/ccr3.7826

**Published:** 2023-08-24

**Authors:** Alwaleed Al‐Dairy, Reem Ahmad, Rawan Hasan

**Affiliations:** ^1^ Cardiac Surgery, Faculty of Medicine Damascus University Damascus Syria; ^2^ Faculty of Medicine Damascus University Damascus Syria

**Keywords:** intra‐atrial tunnel, persistent left superior vena cava, unroofed coronary sinus

## Abstract

**Key Clinical Message:**

Awareness of persistent left superior vena cava (PLSVC) with unroofed coronary sinus is crucial. Pre‐ and perioperative evaluation of this association is necessary for surgical plan. Creating an intra‐atrial tunnel to divert LSVC to right atrium without obstructing the mitral valve or the pulmonary veins is the safe surgical approach.

**Abstract:**

Unroofed coronary sinus syndrome is a rare congenital heart defect representing less than 1% of all atrial septal defect (ASD) types, and may be associated with persistent left superior vena cava (PLSVC) which may be missed during preoperative diagnosis. Herein, we present a case of a 2‐year‐old patient who underwent an operation for repair of a coronary sinus‐type ASD; however, PLSVC was detected intraoperatively. An antra‐atrial tunnel has created to divert the flow of PLSVC into the right atrium along with the repair of the ASD.

## Graphical abstract

Misdiagnosis of PLSVC with unroofed coronary sinus.

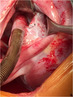

## 1 INTRODUCTION

Persistent left superior vena cava (PLSVC) is the most common thoracic venous anomaly, with an incidence of 0.2% in normal neonates.^1^ However, it is more common among patients with congenital heart diseases (CHD) (1.4%), and recognition of its presence by preoperative diagnosis is of great importance during congenital heart surgery.^1^, ^2^, ^3^, ^4^ Commonly, PLSVC drains into the right atrium (RA) via the coronary sinus (CS), and when isolated, it is usually asymptomatic, and may be detected incidentally.^1^ Unroofed coronary sinus syndrome (UCSS) is an uncommon CHD which leads to a left to right shunt at the atrial level, and comprises <1% of all atrial septal defect (ASD) types.^2^, ^5^ When UCSS is associated with PLSVC, it is difficult to detect the diagnosis by transthoracic echocardiography (TTE).^1^ Herein, we present a case of a 2‐year‐old patient with preoperative diagnosis of coronary sinus‐type ASD; however, PLSVC draining into the left atrium (LA) was detected during surgery.

## 2 CASE PRESENTATION

**FIGURE 1:**
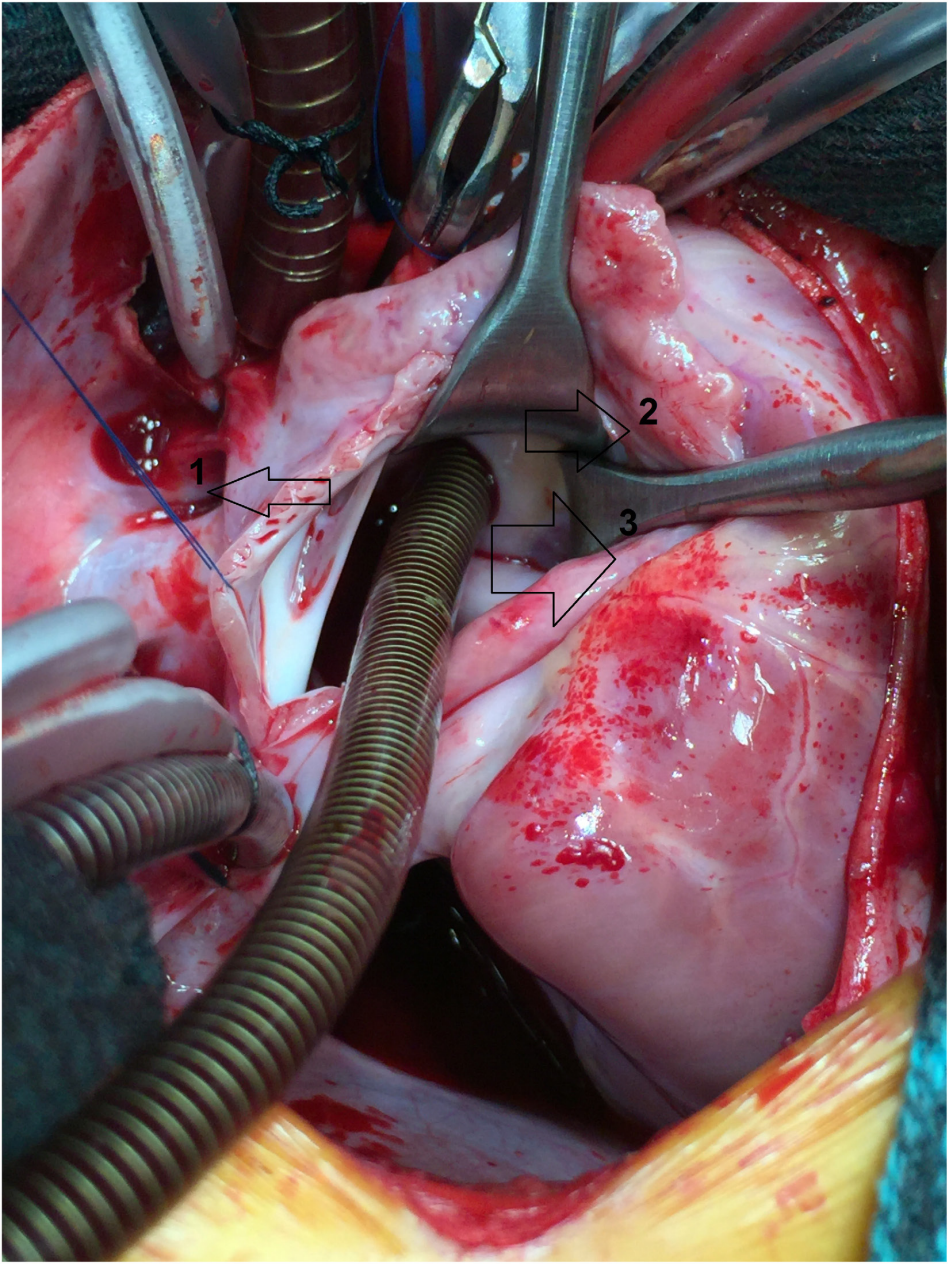
Intraoperative image showing the venous cannula placed through the atrial septal defect toward persistent left superior vena cava (PLSVC) orifice. 1: The opened right atrium; 2: left atrium; 3: the venous cannula placed into PLSVC orifice.

**FIGURE 2:**
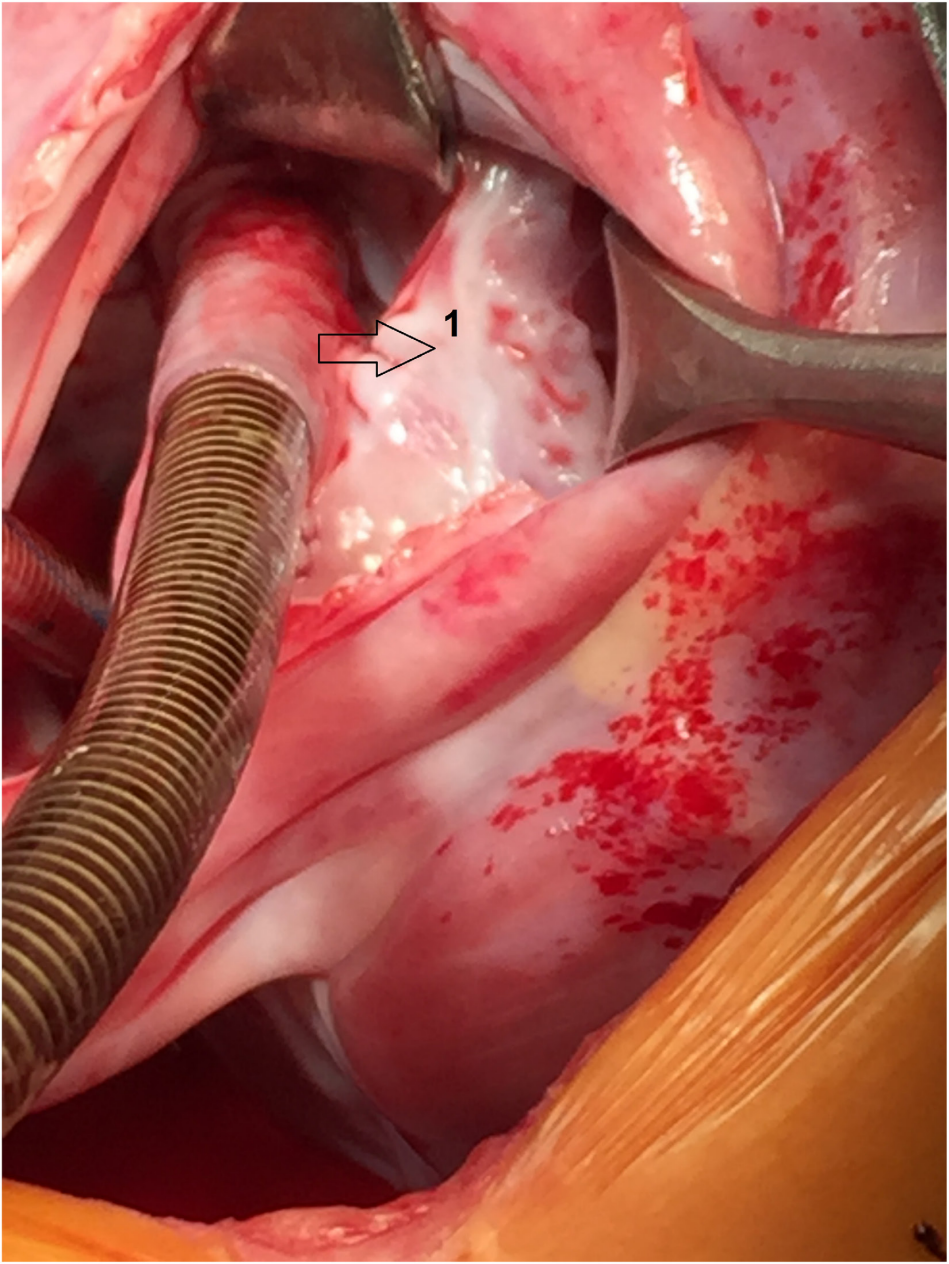
Intraoperative image showing the intra‐atrial tunnel. 1: the pericardial patch used to construct the tunnel.

**FIGURE 3:**
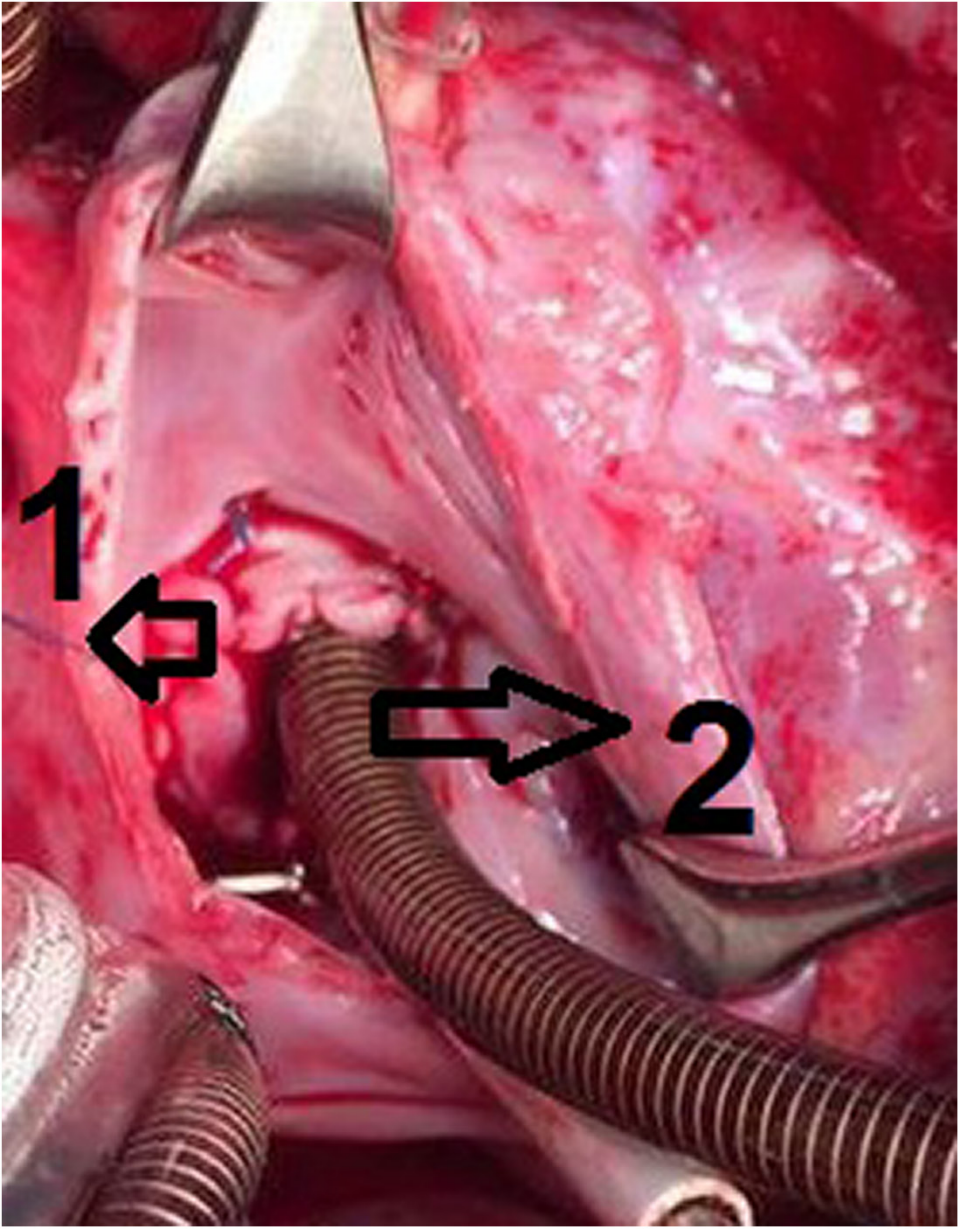
Intraoperative image showing the final anatomy after closing the atrial septal defect (ASD). 1: the pericardial patch used for ASD closure; 2: the venous cannula placed in the intra‐atrial tunnel to divert persistent left superior vena cava flow into right atrium.

A 2‐year‐old girl was presented to our hospital when her parents noticed that she suffered from moderate tachypnea recently. Physical examination revealed a 3/6 systolic murmur on the left sternal border and there was not any cyanosis. TTE showed an ASD of coronary sinus type of about 1.5 cm with dilated right heart cavities. The patient was scheduled for surgical closure of the ASD. Upon anesthesia, oxygen saturation (SaO_2_) was 100%, and the operation was performed through median sternotomy. The pericardium was opened, and PLSVC was noticed. Complete cardiopulmonary bypass was prepared without cannulating the PLSVC. The heart was arrested by antegrade cold blood cardioplegic solution. The RA was opened with an incision parallel to the right atrioventricular groove as usual. On inspection, we found that the PLSVC was draining into the roof of the LA, and a venous cannula was directed through the ASD toward its orifice to drain it (Figure 1). The pulmonary vein drainage and the mitral valve were inspected. A fresh autologous pericardial patch was used to construct a tunnel that drains the PLSVC into RA (Figure 2). The ASD was closed by another fresh autologous pericardial patch and thus the PLSVC will drain into RA (Figure 3). The remainder of the operation was completed uneventfully.

## 3 DISCUSSION

UCSS is a rare CHD representing less than 1% of all ASD types.^1^ Its clinical manifestation is atypical, and may be associated with varied CHDs.^6^ Preoperative diagnosis by TTE is very difficult when associated with PLSVC.^2^, ^6^ In our patient, the diagnosis of associated PLSVC was missed preoperatively, and was detected intraoperatively, and this had led to a change in the surgical plan. Our surgical approach was to create an intra‐atrial tunnel to divert the flow of PLSVC into RA with great attention to the pulmonary veins and the mitral valve, and repair the ASD with fresh autologous pericardial patch. Other operative options include the following: ligating PLSVC and repairing the ASD; constructing a baffle to guide PLSVC toward RA.^6^ We confirm the importance of precise preoperative diagnosis to avoid any complications from such an incidental intraoperative finding.

## 4 CONCLUSION

UCSS is a rare CHD; however, its association with PLSVC must be kept in mind. Creating an intra‐atrial tunnel to divert the flow of PLSVC into RA, without obstructing the mitral valve or the pulmonary veins, is a safe surgical approach.

## AUTHOR CONTRIBUTIONS

**Alwaleed Al‐Dairy:** Conceptualization; data curation; investigation; project administration; resources; software; supervision; writing – original draft; writing – review and editing. **Reem Ahmad:** Data curation; writing – original draft; writing – review and editing. **Rawan Hasan:** Data curation; writing – original draft; writing – review and editing.

## FUNDING INFORMATION

There is no funding resource for writing this manuscript.

## CONFLICT OF INTEREST STATEMENT

The authors have no conflict of interest.

## ETHICS STATEMENT

The manuscript was approved by the ethics committee at Damascus University.

## CONSENT

Written informed consent was obtained from the patient's parents for publication of this report and the images.

## Data Availability

The data that support the findings of this study are available from the corresponding author, [A.A], upon reasonable request.
